# An Efficient Protocol for the Green and Solvent-Free Synthesis of Azine Derivatives at Room Temperature Using BiCl_3_-Loaded Montmorillonite K10 as a New Recyclable Heterogeneous Catalyst

**DOI:** 10.5402/2012/595868

**Published:** 2012-10-08

**Authors:** K. Ravi, B. Krishnakumar, M. Swaminathan

**Affiliations:** Photocatalysis Laboratory, Department of Chemistry, Annamalai University, Annamalainagar 608 002, Tamil Nadu, India

## Abstract

A new BiCl_3_-loaded montmorillonite K10 catalyst has been prepared by solid dispersion method and was characterized by X-ray diffraction (XRD), field emission scanning electron microscopy (FE-SEM), and cyclic voltammetry (CV) measurements. BiCl_3_ loaded K10 (BiCl_3_-K10) has been used as solid acid catalyst for the synthesis of azine derivatives from benzophenone hydrazone and ketones/aldehydes by simple physical grinding. This BiCl_3_-K10 gives an excellent yield with short reaction time and is an inexpensive, easily recyclable catalyst for this reaction.

## 1. Introduction

Commercially available BiCl_3_ had been widely used as a Lewis acid catalyst for aldol reaction [1], hetero Diels-Alder reaction [2], ring opening of epoxides with aromatic amines [3], deoxygenative allylation of substituted benzylic alcohols with allyltrimethylsilane [4], and for three-component synthesis of *β*-amino carbonyl compounds [5]. But BiCl_3_ is highly hygroscopic and difficult to handle as it is toxic and causes irritation to the gastrointestinal and respiratory tract. In addition, this catalyst cannot be reused. Bismuth chloride is to be loaded on a support material for easy handling and utilization.

Clays function as efficient catalysts for various organic transformations due to their Bronsted and Lewis acidities in their natural and ion-exchanged forms [6]. Commercially available montmorillonite K10 (Mont K10) clay is one such material that can fulfill these requirements. Mont K10 is an environmentally benign and economically feasible solid catalyst that offer several advantages, such as ease of handling, non-corrosiveness, low cost, and regeneration. Its high surface area (250 m^2 ^g^−1^) makes it as a useful and active catalyst. Its structural feature [7] and synthetic potential [8] have been extensively studied. Mont K10 is a layered alumino-silicate with a dioctahedral layer sandwiched between two tetrahedral layers. Due to strong catalytic activity as solid acid, Mont K10 clay has been used extensively as a catalyst for many organic transformations [9–12].

Azines, R_1_R_2_C=N–N=CR_1_R_2_, have attracted great attention in organic synthesis as they are good synthons for obtaining various heterocyclic compounds [13, 14]. These compounds constitute an important class of compounds with unexpected biological activities [15, 16]. The usual method for the preparation of azines involves treatment of carbonyl compounds with hydrazine hydrate and acetic acid in ethanol [17]. A number of methods have been reported for the synthesis of azines under various conditions [18–21], but most of them require elevated temperatures and complex catalysts. Hence, there is a need to develop a simple, eco-friendly method under mild conditions for the preparation of azines. Recent research is mainly focused on the use of environmental benign catalysts under solvent free conditions [22, 23]. In continuation of work on the synthesis of azine derivatives with sulfated titania under solvent free conditions [24], herein we report a recyclable, easily separable, eco-friendly, and highly effective catalytic system BiCl_3_ loaded K10 (BiCl_3_-K10) for the synthesis of azines derivatives in excellent yield by simple physical grinding. To the best of our knowledge this is the first report of azine derivatives in this catalytic way using BiCl_3_-K10.

## 2. Results and Discussion

### 2.1. Characterization of BiCl_3_-K10

**Figure 1:**
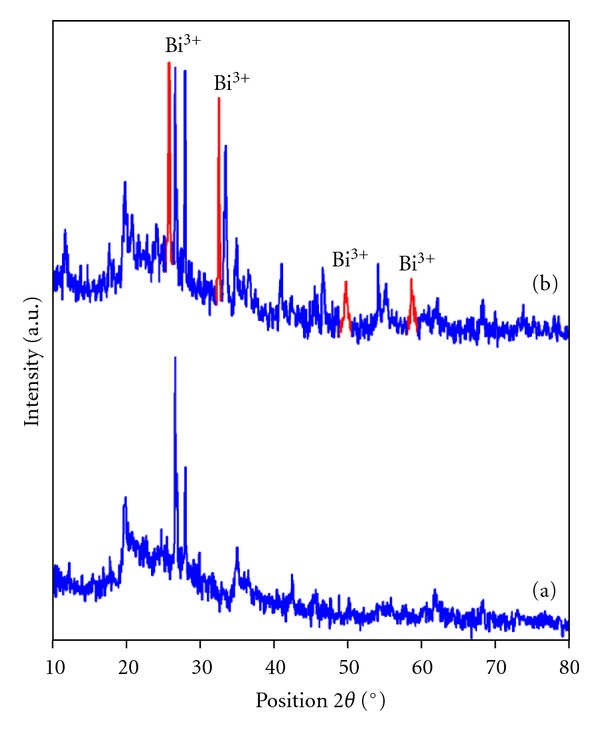
XRD of (a) Mont K10 and (b) BiCl_3_-K10.

**Figure 2:**
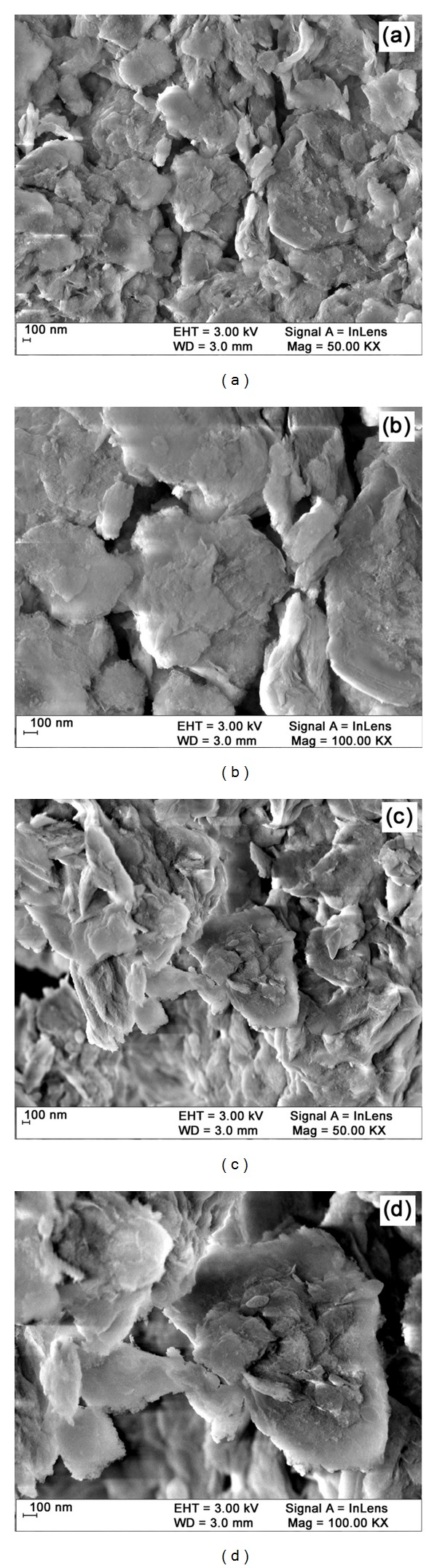
FE-SEM images of (a) Mont K10 (50 KX), (b) Mont K10 (100 KX), (c) BiCl_3_-K10 (50 KX), and (d) BiCl_3_-K10 (100 KX).

BiCl_3_ loaded K10 has been characterized by XRD, SEM, and CV measurements. XRD analysis of the dried montmorillonite K10 and BiCl_3_-K10 catalyst are shown in Figures 1(a) and 1(b), respectively. The peaks observed at 26.6°, 45.5°, and 54.8° are attributed to montmorillonite (Figure 1(a)) [25]. The XRD pattern of BiCl_3_-K10 is different from that of montmorillonite K10 (Figure 2(b)). In the BiCl_3_-K10 system, four new peaks obtained with 2*θ* values of 25.8°, 32.4°, 49.6°, and 58.5° corresponding to Bi^3+^ confirms the loading of BiCl_3_ [26]. 

**Figure 3:**
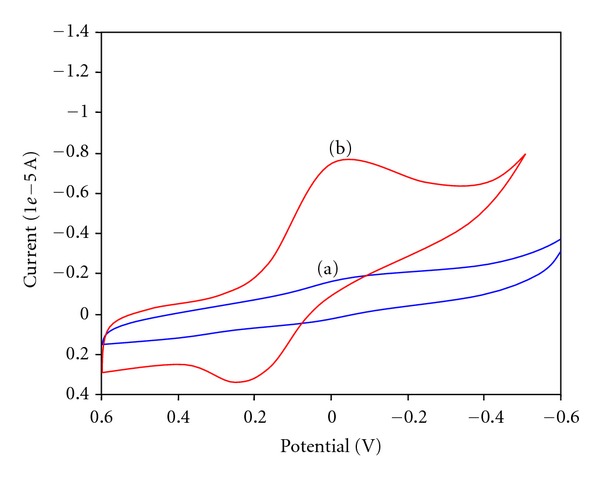
Cyclic voltammograms of (a) Mont K10 and (b) BiCl_3_-K10.

The structure and morphology of the catalyst are very important parameters as they influence the catalytic activity. The surface morphology of catalysts has been analyzed by FE-SEM images. The FE-SEM images different locations of each catalyst are given in Figure 2. FE-SEM images at two different magnifications with different locations of BiCl_3_-K10 reveal that the particles are agglomerated by Bi loading (Figures 2(c) and 2(d)) when compared to pure K10 (Figures 2(a) and 2(b)). In cyclic voltammetry measurements, pure K10 and BiCl_3_-K10 were used in the redox reaction of potassium ferrocyanide (3 mM concentration). This measurement is used to find out the electrical conductivity of the catalyst. A predetermined amount of catalyst was dispersed in a 0.1% nafion in ethanol solution for 1 h in an ultrasonic bath to form a stable suspension. The catalyst nanocomposite was deposited on the glassy carbon electrode by droplet evaporation for 15 min and then drying in nitrogen atmosphere for 20 min. Bare K10 did not give any anodic potential and current (Figure 3(a)) but BiCl_3_ loaded K10 gave anodic potential and current of 0.212 V and 2.50 × 10^−6^ A, respectively (Figure 3(b)). This increase in current indicates presence of “Bi” in the catalyst [27]. 

### 2.2. Synthesis of 1-(Diphenylmethylidene)-2-(1-Phenylethylidene)hydrazine, **3a**

To a mixture of benzophenone hydrazone (1 mmol) and acetophenone (1 mmol) in dry media, 0.1 g of BiCl_3_-K10 was added and the mixture was taken in a mortar and ground with a pestle at room temperature for 1.5 min. Completion of the reaction was tested by thin layer chromatography (TLC). After completion of the reaction, ethyl acetate was added to the solidified mixture and the insoluble catalyst was separated by filtration. The filtrate was dried over anhydrous Na_2_SO_4_. The solvent was evaporated to get the product. Then it was subjected to GC and GC-MS analysis for the determination of the product yield. The structure of product obtained was confirmed by FT-IR, ^1^H, ^13^C NMR, and GC-MS analysis. Simple physical grinding did not give the product for the condensation of benzophenone hydrazone with benzophenone.

*1-(Diphenylmethylidene)-2-(1-phenylethylidene)hydra*-*zine,* **3a**. m.p. = 105–107°C; IR (KBr) (cm^−1^) = 3053, 3024, 2926, 2838, 1595, 1563, 1441, 1361, 1295, 782, 694; ^1^H NMR (CDCl_3_, 400 MHz) (**δ**, ppm) = methyl proton 2.31 (s, 3H), other aromatic protons 7.11–7.62 (m, 15H); ^13^C NMR (CDCl_3_, 100 MHz) (**δ**, ppm) = methyl carbon 15.6 and other aromatic and *ipso* carbons 126.7–138.4 and two C=N 158.8 and 159.6; GC-MS (*m/z*) = 299.2 (M+1).

### 2.3. Preparation of Bis(diphenylmethylidene)hydrazine, **3o**-Procedure

To a mixture of benzophenone hydrazone (1 mmol) and benzophenone (1 mmol) in dry media, 0.1 g of BiCl_3_-K10 was added and the mixture was irradiated under microwave oven at 480 W for 6 min. Completion of the reaction was tested by TLC. After completion of the reaction, product was separated by the same procedure given above and subjected to GC and GC-MS analysis for the determination of the yield of the product. The structure of product was confirmed by FT-IR, ^1^H, ^13^C NMR, and GC-MS analysis.

*Bis(diphenylmethylidene)hydrazine,* **3o**. m.p. = 163–165°C; IR (KBr) (cm^−1^) = 3079, 3054, 1638, 1564, 1487, 1442, 1318, 767, 659; ^1^H NMR (CDCl_3_, 300 MHz) (**δ**, ppm) = aromatic protons 7.28–7.53 (m, 20H); ^13^C NMR (CDCl_3_, 300 MHz) (**δ**, ppm) = aromatic carbons 127.8, 128.0, 128.6, 128.7, 129.3, 129.6, 135.5, 138.2, and C=N 158.9; GC-MS (*m/z*) = 361.2 (M+1).

 Spectral data of the some selected compounds are given below.

*1-Benzylidene-2-(Diphenylmethylidene)hydrazine,* **3f**. m.p. = 97–98°C; IR (KBr) (cm^−1^) = 3055, 3025,1610, 1569, 1444, 1317, 765, 692; ^1^H NMR (CDCl_3_, 400 MHz) (**δ**, ppm) = N=C–H proton 8.62 (s, 1H), other aromatic protons 7.22–7.82 (m, 15H); ^13^C NMR (CDCl_3_, 100 MHz) (**δ**, ppm) = C–H carbon 96.18 and other aromatic and *ipso* carbons 128.1–159.2 and C=N 161.8; GC-MS (*m/z*) = 285.1 (M+1).

*1-(4-Bromobenzylidene)-2-(diphenylmethylidene)hydra*-*zine,* **3k**. m.p. = 103–105°C; IR (KBr) (cm^−1^) =3053, 2978, 2919, 1602, 1560, 1483, 1442, 1320, 1297, 776, 524; ^1^H NMR (CDCl_3_, 400 MHz) (**δ**, ppm) = N=C–H proton 8.45 (s, 1H), other aromatic protons 7.27–7.69 (m, 14H); ^13^C NMR (CDCl_3_, 100 MHz) (**δ**, ppm) = C–H carbon 96.18 and other aromatic and *ipso* carbons 125.1, 127.5, 128.1, 128.9, 129.2, 129.7, 130.2, 131.8, 133.5, 135.5, 138.1, 157.8, and C=N 166.2; GC-MS (*m/z*) = 364.1 (M+1).

### 2.4. Effect of Operational Parameters

**Scheme 1:**
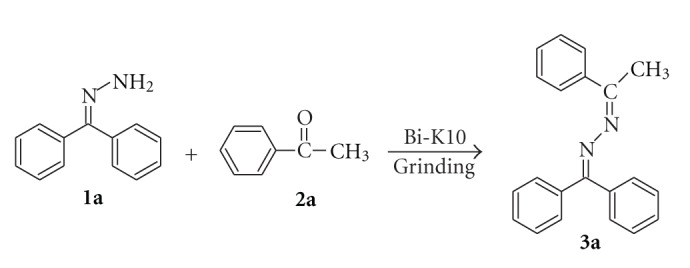
The condensation reaction of benzophenone hydrazone **1a** with acetophenone **2a **catalyzed by BiCl_3_-K10.

**Scheme 2:**
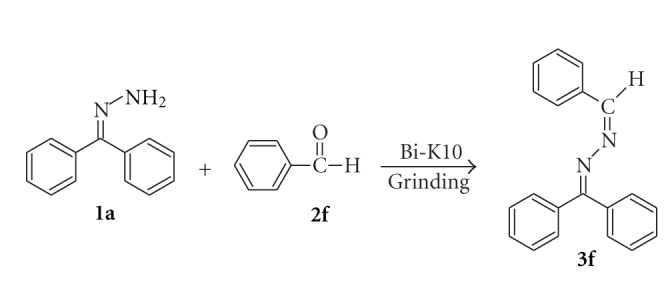
The condensation reaction of benzophenone hydrazone **1a** with benzaldehyde **2f** catalyzed by BiCl_3_-K10.

When a mixture of benzophenone hydrazone (1 mmol) and acetophenone (1 mmol) without catalyst was ground for 3 min no reaction occurred. However, grinding the mixture with 0.1 g of BiCl_3_-K10 initiated a condensation reaction producing 98% 1-(diphenylmethylidene)-2-(1-phenylethylidene)hydrazine (**3a**) in 1.5 min (Scheme 1). The above reaction was carried out by various catalysts (Table 1). When bare Mont K10 was used in the same reaction only 80% of product was obtained. BiCl_3_-K10 is found to more efficient in less reaction time when compared to other catalysts. With benzaldehyde corresponding azine was produced (Scheme 2). 

**Figure 4:**
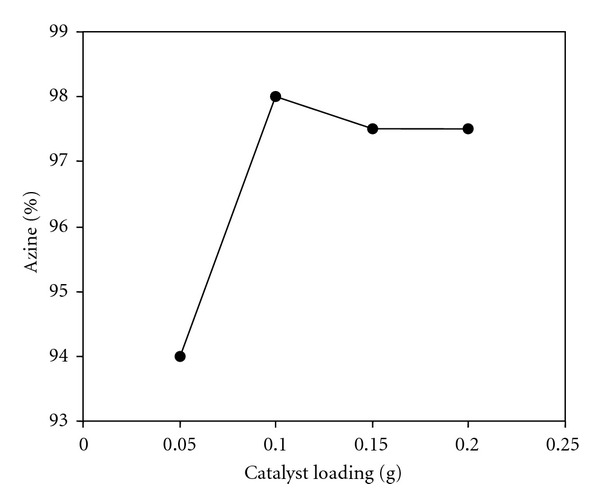
Effect of catalyst loading. Catalyst = BiCl_3_-K10, benzophenone hydrazone = 1 mmol, acetophenone = 1 mmol, time = 1.5 min (grinding at solvent free condition in room temperature).

The effect of catalyst (BiCl_3_-K10) dosage on the formation of azine **3a** was investigated by varying the catalyst amount from 0.05 to 0.2 g (Figure 4). When the amount of the catalyst is increased from 0.05 to 0.1 g, azine formation increases from 94.0 to 98.0%. This is due to increase in the number of catalyst particles. Above 0.1 g of the catalyst, no significant change in the percentage of product formation occurred. The optimum catalyst loading is found to be 0.1 g for the conversion of 1 mmol of benzophenone hydrazone. 

### 2.5. Synthesis of Substituted Azines

In order to show the generality and scope of this new protocol, we used various substituted ketones and aldehydes for condensation with benzophenone hydrazone and the results obtained are summarized in Table 2. Condensation with all aldehydes and ketones (except benzophenone; Table 2, entry 15) proceeded very cleanly at room temperature and no undesirable side-reactions were observed. Yields were not much affected by the substituents present in the ketones and aldehydes (Table 2, entries 1–12). Furfuraldehye and pyridine-2-aldehyde also gave good yield (Table 2, entries 13 and 14). Overall, aldehydes react faster than ketones with benzophenone hydrazone.

**Scheme 3:**
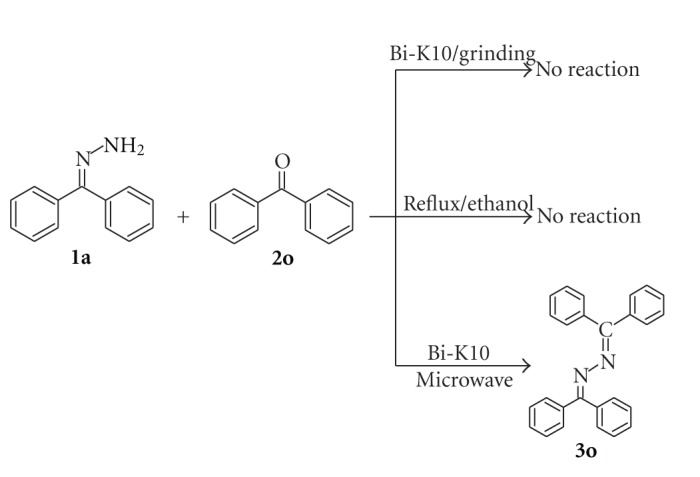
The condensation reaction of benzophenone hydrazone **1a** with benzophenone **2o **catalyzed by BiCl_3_-K10.

However, in the case of benzophenone, no reaction was observed when it was ground with benzophenone hydrazone at room temperature for 10 min in the presence of BiCl_3_-K10. No product was obtained even by refluxing the mixture in alcohol (Scheme 3). This may be due to steric effect of two phenyl groups present in the benzophenone. This steric effect is also reflected with benzaldehyde and acetophenone. Benzaldehyde reacts faster than acetophenone with benzophenone hydrazone. Hence, synthesis of benzophenone azine was carried out under microwave irradiation. The use of microwaves in organic synthesis has attracted considerable attention in recent years due to less reaction time and improved product yield [28, 29]. 

When a mixture of benzophenone hydrazone (1 mmol) and benzophenone (1 mmol) without solvent was irradiated in microwave oven (480 W) for 10 min, no reaction was observed. However, addition of a 0.1 g of BiCl_3_-K10 to this mixture has initiated condensation reaction producing 98% 1,2-bis(diphenylmethylene)hydrazine in 6 min under microwave irradiation (Scheme 3) (Table 2, entry 15). Structure of this product has been confirmed by FT-IR, ^1^H NMR, ^13^C NMR, and GC-MS data. The synthesis of azines at room temperature by sulfated anatase-titania was reported earlier from our laboratory [30]. It was found that formation of 1,2-bis(diphenylmethylene)hydrazine with sulfated titania under microwave oven required 8 min whereas in the case of BiCl_3_-K10, this product was obtained in 6 min. Furthermore, the preparation of BiCl_3_-K10 is simple, when compared to sulfated anatase-titania.

**Scheme 4:**
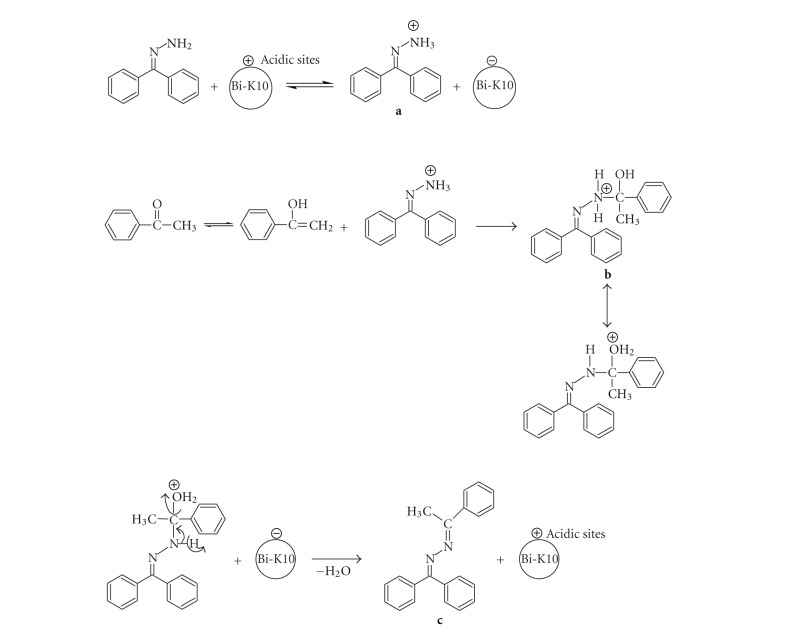
Proposed mechanism for the condensation reaction of benzophenone hydrazone **1a** with acetophenone **2a** catalyzed by BiCl_3_-K10.

Since BiCl_3_-K10 acts as a solid acid catalyst, acid catalyzed mechanism is proposed for this reaction (Scheme 4). This mechanism involves the protonation of hydrazone with acidic BiCl_3_-K10. This protonated hydrazone **(a)** condenses with enolic form of the ketone forming an intermediate **(b)** which on dehydration and deprotonation produces the product azine **(c)**. Solid acid catalyst BiCl_3_-K10 promotes dehydration and deprotonation. Any heterogeneous reaction, The advantage of this heterogeneous reaction is its reusability. The possibility of recycling the catalyst was examined for the reaction of benzophenone hydrazone with acetophenone. When the reaction was complete, ethyl acetate was added to the solidified mixture and the insoluble catalyst was separated by filtration. The separated catalyst was dried under hot air oven at 100°C for 5 h and could be used five times without appreciable loss in its catalytic activity up to fifth run (94.0%) (Table 3, entry 5).

## 3. Experimental

### 3.1. Materials and Methods

Acetophenone, substituted acetophenones and bismuth chloride (s.d. Fine), benzaldehyde, substituted benzaldehydes, furfuraldehyde, pyridine-2-aldehyde, and hydrazine hydrate (Aldrich Chemicals) were used as received. Montmorillonite K10 clay obtained from Aldrich, has the following chemical composition (wt%) SiO_2_: 67.6; Al_2_O_3_:  14.6; Fe_2_O_3_:  2.9; MgO:  1.8. The benzophenone hydrazone was prepared according to the literature procedure [17].

### 3.2. Apparatus

IR spectra were recorded using an Avatar-330 FT-IR spectrophotometer using KBr pellets. For GC analysis, a Perkin-Elmer GC-9000 with a capillary column of DB-5 and flame ionization detector was used. GC-MS analysis was carried out using a GC model: Varian GC-MS-Saturn 2200 Thermo, capillary column VF5MS (5% phenyl–95% methylpolysiloxane), 30 m length, 0.25 mm internal diameter, 0.25 *μ*m film thickness, temperature of column range from 50 to 280°C (10°C  min⁡^−1^), and injector temperature 250°C. Proton and carbon NMR spectra were recorded on a BRUKER AVIII FT-NMR spectrometer operating at 400 MHz for all the samples. 

### 3.3. Preparation of BiCl_3_-K10 Catalyst

**Scheme 5:**
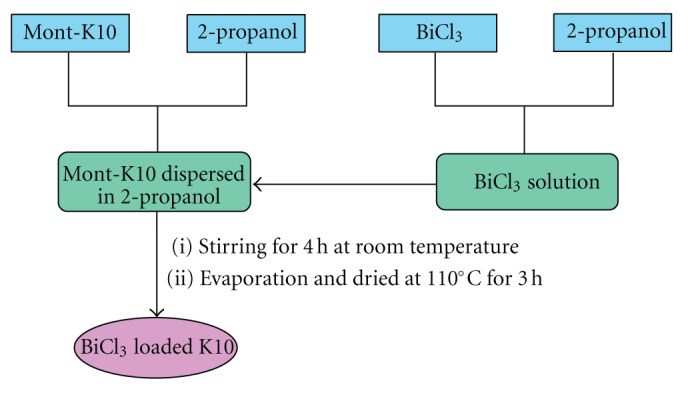
Preparation of BiCl_3_-K10 solid acid catalyst.

A new BiCl_3_-K10 was prepared by simple solid dispersion method (Scheme 5). 2.7 g of montmorillonite K10 clay was dispersed in 50 mL of 2-propanol. 2.5 × 10^−4^ M of BiCl_3_, (0.081 g) is dissolved in 10 mL 2-propoanol and added to Mont K10-2-propanol mixture. The mixed suspension was stirred for 4 h at room temperature was evaporated to obtain the product. Then it was dried at 110°C for 3 h. The BiCl_3_-K10 catalyst was obtained as a fine powder. This catalyst contains 3 wt% of Bi.

## 4. Conclusions

A new BiCl_3_ loaded K10 was prepared by simple solid dispersion method at room temperature and was characterized by XRD, FE-SEM, and CV measurements. Presence of Bi^3+^ was confirmed by XRD and CV measurements. BiCl_3_-K10 is introduced as an excellent catalytic system for the synthesis of azine derivatives by simple grinding at room temperature. This novel and practical method has the advantages of mild conditions, excellent yield of products with short reaction time. BiCl_3_-K10 is found to be reusable.

## Figures and Tables

**Table 1:** Condensation of benzophenone azine (1 mmol) with acetophenone (1 mmol) using various catalysts at room temperature.

Entry	Catalyst	Grinding time (min)	% of azine^a^
1	TiO_2_ (anatase)	5	Trace
2	TiO_2_-P25	5	Trace
3	Sulfated TiO_2_-P25	5	20
4	ZrO_2_	5	Trace
5	Sulfated ZrO_2_	5	15
6	ZnO (Merck)	5	Trace
7	TiO_2_ (Merck)	5	Trace
8	Mont K10	5	80
9	BiCl_3_-K10	2	98

^a^Yields with respect to benzophenone hydrazone.

**Table 2:** Synthesis of azine derivatives using Bi-K10 under solvent-free conditions.

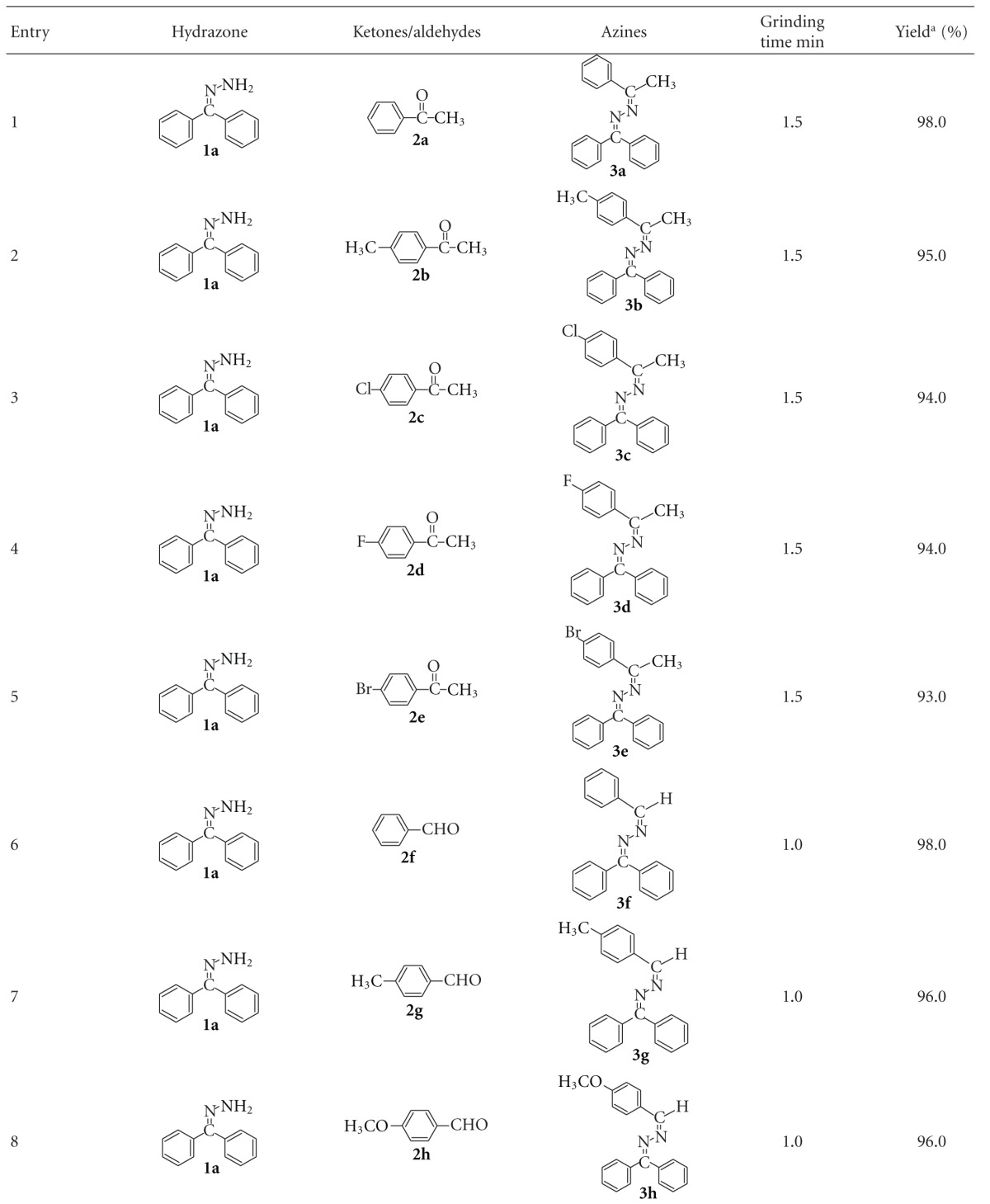 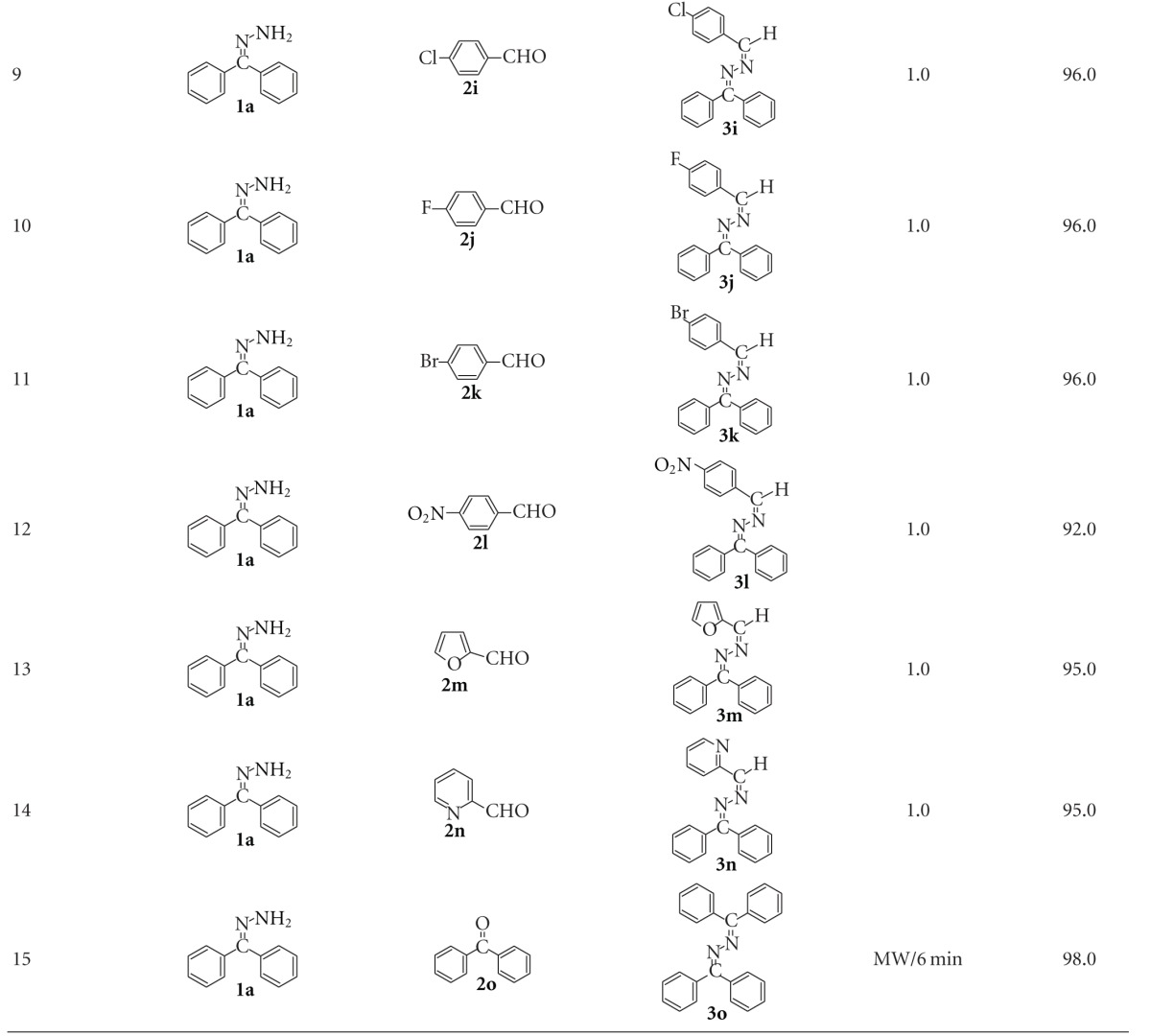

^a^Yields with respect to hydrazone.

**Table 3:** Reusability of Bi-K10 on condensation of benzophenone hydrazone (1 mmol) and acetophenone (1 mmol) under grinding at room temperature.

Entry	1	2	3	4	5
Yield^a^	98.0	96.0	95.0	94.0	94.0

^a^Yields with respect to benzophenone hydrazone.
